# The who, where and how of fusobacteria and colon cancer

**DOI:** 10.7554/eLife.28434

**Published:** 2018-03-13

**Authors:** Cynthia L Sears

**Affiliations:** Bloomberg-Kimmel Institute for ImmunotherapyJohns Hopkins University School of MedicineBaltimoreUnited States

**Keywords:** Reproducibility Project: Cancer Biology, replication, metascience, reproducibility, Fusobacterium nucleatum, colorectal carcinoma, Human

## Abstract

The association between the bacterium *Fusobacterium nucleatum* and human colon cancer is more complicated than it first appeared.

**Related research article** Repass J, Reproducibility Project: Cancer Biology. 2018. Replication Study: *Fusobacterium nucleatum* infection is prevalent in human colorectal carcinoma. *eLife* **7**:e25801. doi: 10.7554/eLife.25801

Trillions of microbes live on or in the body of a typical human, and this relationship is mostly harmonious. Our colons harbor the highest density of bacteria, with a mucus barrier protecting our gut from them. For 50 years, scientists have researched who amongst these bacteria might cause colon cancer, where these bacteria might act in the colon, and how they might cause colon cancer.

In 2012 researchers at the BC Cancer Agency, Simon Fraser University and the University of Guelph reported that they had used sequence-based technologies to show that *Fusobacterium nucleatum*, a species not previously linked to cancer, might be associated with colon cancer (Castellarin et al., 2012). They found that the levels of *F. nucleatum* in colorectal carcinoma were significantly higher than the levels in adjacent normal tissue. An independent group also reported a similar finding at the same time (Kostic et al., 2012). This was a surprise as *F. nucleatum* is usually found in the mouth.

In 2016, as part of the Reproducibility Project: Cancer Biology, Repass et al. published a Registered Report which explained in detail how they would seek to replicate the experiment (figure 2 in Castellarin et al.) in which quantitative polymerase chain reaction (qPCR) was used to detect *F. nucleatum* in colon tissues taken from colon cancer patients (Repass et al., 2016). The results of this experiment have now been published as a Replication Study (Repass and Reproducibility Project: Cancer Biology, 2018). In short, *F. nucleatum* was detected in just 25% of colorectal carcinomas in the Replication Study, and the difference in the level of this species in colorectal carcinomas and adjacent tissues (there was 10% more *F. nucleatum* in the carcinomas) was not significant.

So why did the Replication Study not find what was reported in the original study? One possible explanation is that Castellarin et al. studied 99 colon tissue pairs (tumor and tumor-adjacent tissues) from colon cancer patients. The Replication Study, on the other hand, included samples from 40 patients with cancer (both tumor and adjacent tissue), along with 40 non-diseased control tissue samples from age, sex and ethnicity matched individuals. A power calculation had suggested that a sample size of 40 would be big enough to see the effect reported in the original paper, assuming that the two populations were clinically similar. However, Castellarin et al. did not fully report the clinical metadata for their study (such as age, gender, ethnicity and risk factors for disease), so differences between the populations might explain why the original results were not replicated. This possibility is also supported by the fact that the relative abundance of *F. nucleatum* in tumors in the original experiment is much higher than in the Replication Study.

Technical issues might also have contributed to the lack of replication: in particular, in the Replication Study, qPCR suggested that *F. nucleatum* DNA was present in 26 samples, albeit at low levels, but qPCR amplicon sequencing detected the presence of specific *F. nucleatum* gene products in just 16 of these samples (10 in carcinomas; 6 in adjacent tissues). Moreover, all the non-specific amplicons (that is, the 10 that were not due to *F. nucleatum*) were detected very close to the detection limit of this technique.

Given that numerous studies have already shown that there is an association between *F. nucleatum* and the microbiota of human colon cancer (see Repass et al., 2016 and Gholizadeh et al., 2017 for reviews), the Replication Study is not a reason to change our view of this association. Rather, it provides a critical opportunity to reflect on our growing, yet incomplete, knowledge regarding fusobacteria and colon cancer. First, and most obvious, there is a need for prospective human studies in well-defined populations – using both microbiology and bioinformatics approaches – to carefully probe how risk factor and other clinical features might alter colon health in the presence of fusobacteria. For example, while *F. nucleatum* has certainly been the species most consistently identified in association with colon cancer to date, many studies have raised the possibility that other species of fusobacteria, or bacteria that co-aggregate in the presence *F. nucleatum*, could contribute to the pathogenesis of colon cancer (Castellarin et al., 2012; Kostic et al., 2012; Drewes et al., 2017; Bullman et al., 2017).

Further, while it is clear that fusobacteria are highly genetically diverse (Castellarin et al., 2012; Manson McGuire et al., 2014), we do not as yet understand whether the strains associated with colon cancer exhibit unique genetic features or are related to the fusobacteria that are common in the mouth.

There are also other gaps in our understanding. For example, some experiments that used daily inoculations of *F. nucleatum* in mouse models of colon cancer suggest that *F. nucleatum* acts early in colon cancer (Kostic et al., 2013; Yang et al., 2017), whereas germ-free mouse experiments – which are, arguably, a more definitive way to test the role of *F. nucleatum* on its own in tumor induction – refute this result (Tomkovich et al., 2017). In contrast, at least two studies (Castellarin et al., 2012; Bullman et al., 2017) suggest that *F. nucleatum* acts late in the tumor process, and research by the present author and co-workers could not discern an association with tumor stage (Drewes et al., 2017).

There is also uncertainty about where *F. nucleatum* is found: some researchers have reported that it is found with increased frequency in cancers on the right side of the colon (Mima et al., 2016), whereas this effect was not seen in other experiments (Drewes et al., 2017). The question of 'where?' is also complicated by the fact that right colon cancer contains prominent mucus-invasive bacterial biofilms, and about one third of these exhibit blooms of *F. nucleatum*.

Lastly, we lack a clear picture of how *F. nucleatum* contributes to the emergence of human colon cancer. Limited data suggest a wide range of putative mechanisms (including Wnt nuclear signaling, immune cell recruitment, checkpoint molecules and specific miRNA induction). While each mechanism is plausible, we lack a cohesive, step-by-step story for *F. nucleatum* carcinogenesis (Gholizadeh et al., 2017; Kostic et al., 2013; Yang et al., 2017).

While the Replication Study did not replicate the results of Castellarin et al., it provided useful information about the importance of population differences and the need for accurate *F. nucleatum* detection methods, and highlighted how much we need to learn about the links between *F. nucleatum* and colon cancer. A better understanding of the who (which *Fusobacterium* species and/or associates), the where (where in the colon, where in the world) and the how (which disease mechanisms) will help with the development of new prevention approaches, diagnostics and/or therapies for a cancer that is increasing in the young and also across the globe.

## Note

Cynthia L Sears was the Reviewing Editor for the Registered Report (Repass et al., 2016) and the Replication Study (Repass and Reproducibility Project: Cancer Biology, 2018).
